# Left Bundle Branch Area Pacing to Overcome Coronary Sinus Anatomy-Related Technical Problems Encountered during Implantation of Biventricular CRT—A Case Report

**DOI:** 10.3390/jcm13113307

**Published:** 2024-06-04

**Authors:** Jędrzej Michalik, Roman Moroz, Marek Szołkiewicz, Alicja Dąbrowska-Kugacka, Ludmiła Daniłowicz-Szymanowicz

**Affiliations:** 1 Department of Cardiology and Interventional Angiology, Kashubian Center for Heart and Vascular Diseases, Pomeranian Hospitals, 84-200 Wejherowo, Poland; jedri1616@gmail.com (J.M.); romanmoroz@wp.pl (R.M.); 2 Department of Cardiology and Electrotherapy, Medical University of Gdansk, 80-214 Gdansk, Poland; alidab@gumed.edu.pl (A.D.-K.); ludmila.danilowicz-szymanowicz@gumed.edu.pl (L.D.-S.)

**Keywords:** left bundle branch area pacing, cardiac resynchronization therapy, heart failure, left bundle branch block

## Abstract

The results of clinical trials show that up to one-third of patients who are eligible for cardiac resynchronization therapy (CRT) do not benefit from biventricular pacing. The reasons vary, including technical problems related to left ventricle pacing lead placement in the appropriate branch of the coronary sinus. Herein, we present a case report of a patient with heart failure with reduced ejection fraction and left bundle branch block, in whom a poor coronary sinus bed made implantation of classic biventricular CRT impossible, but in whom, alternatively, rescue-performed left bundle branch area pacing allowed effective electrical and mechanical cardiac resynchronization. The report confirms that left bundle branch area pacing may be a rational alternative in such cases.

## 1. Introduction

Right ventricular pacing (RVP) has been for years a primary pacing modality in patients with atrioventricular conduction disorders, effectively saving the health and life of many patients. Unfortunately, it significantly disturbs originally coordinated and synchronous ventricular electrical activation, triggering harmful myocardial effects, and, eventually, in some patients (approximately 15–20%) leads to the development of pacing-induced cardiomyopathy [1]. Cardiac resynchronization therapy (CRT) was developed to restore and/or maintain a synchronous ventricular depolarization pattern efficiently enough to avoid pacing-induced cardiomyopathy. It was introduced into clinical practice for the first time in 1996. Since that time, it has gained a unique position in cardiac electrotherapy, becoming a mainstay method of pacing in the treatment of patients with symptomatic chronic heart failure and reduced ejection fraction (HFrEF) [2]. The undoubted advantages of CRT are appreciated primarily by patients with intraventricular conduction disorders, especially left bundle branch block (LBBB). The clinical effectiveness of CRT has been confirmed in numerous randomized studies, in which not only a subjective improvement in exercise tolerance and quality of life has been observed, but, above all, a significant reduction in the number of hospitalizations and mortality has been seen [3,4]. Unfortunately, not all patients with HFrEF and co-existing LBBB are the beneficiaries of this therapy. Some patients (30%) fail to respond to CRT via biventricular pacing, and they are considered non-responders to the therapy. Moreover, in some cases there are technical problems that make implantation of biventricular CRT impossible (e.g., poor coronary sinus bed, coronary sinus dissection, unacceptably high left ventricle pacing threshold, or unwanted/unavoidable phrenic nerve stimulation) [5]. In such cases, conduction system pacing (CSP) seems to be an excellent alternative or complementary technique for CRT, even in patients with HFrEF and co-existing LBBB. There are two principle physiological pacing modalities: His bundle pacing, described for the first time in 1999 by Deshmukh et al. [6], and left bundle branch area (LBBAP) pacing, described for the first time in 2017 by Huang et al. [7]. Both methods aim to restore regular electrical activation of the heart via the native conduction system, and they are recognized as methods that can efficiently deliver resynchronization therapy. There is a growing interest in the use CSP to deliver CRT, and the implantation procedure seems to be safe and feasible. One may presume that this form of pacing will dominate cardiac electrotherapy in the coming years. Although there are still no data valuable enough from large clinical trials supporting the efficacy of CSP, or maybe even its superiority over biventricular (BiV) pacing, one cannot ignore the fact that CSP restores and/or maintains a physiological activation of both ventricles and subsequently their synchronous contraction in the most natural, presumably the most optimal, way; intuitively better than any other. In fact, all available research results show that the CSP outcome is at least non-inferior to BiV pacing results [8,9].

## 2. Materials and Methods

This is a report on a patient with symptomatic HFrEF and co-existing LBBB in whom a poor coronary sinus bed made the implantation of biventricular CRT unsuccessful, but LBBAP, performed instead, turned out to be a highly effective alternative for delivering electrical and mechanical resynchronization.

## 3. Case Report

**Figure 1:**
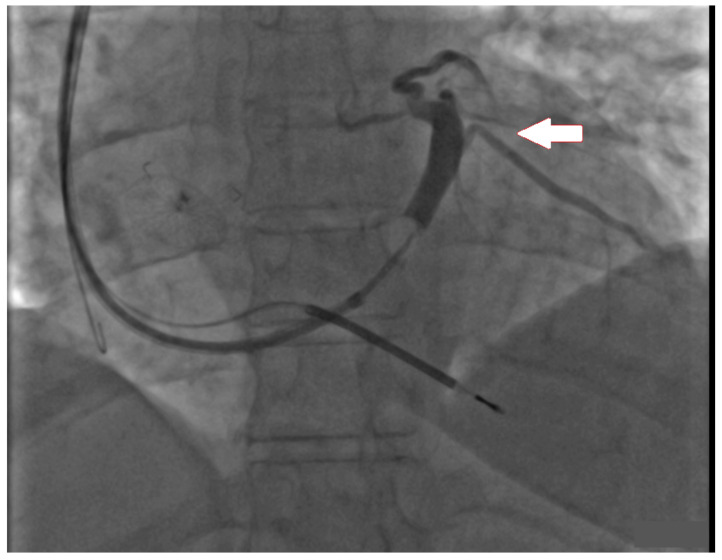
Fluoroscopic image presenting retrograde coronary sinus venography. The arrow indicates the posterolateral vein, which appeared to be quite optimal for left ventricular lead placement, but due to technical problems placement was unachievable.

A 64-year-old woman was primarily admitted to the Department of Cardiology and Interventional Angiology in Kashubian Center for Heart and Vascular Diseases on 31 May 2023, for an elective implantation of CRT with defibrillator (CRT-D). The patient had a number of cardiovascular risk factors, including hypercholesterolemia, hypertension, diabetes, and family history of coronary artery disease. She has never smoked. In April 2022, the patient suffered a non-ST-elevation myocardial infarction (NSTEMI), and was treated with percutaneous angioplasty of the left anterior descending artery with drug-eluting stent implantation. After the event, clinical symptoms of heart failure occurred, and thus European Society of Cardiology (ESC) guidelines-indicated pharmacotherapy was started. In February 2023, the patient experienced another NSTEMI, caused by restenosis in the previously implanted stent. This time, percutaneous coronary intervention with a drug-eluting balloon was performed. There were no significant narrowings in the remaining coronary arteries. Evaluation of the patient performed in June 2023 showed that, despite optimal pharmacological treatment (angiotensin-converting enzyme inhibitor, beta-blocker, aldosterone antagonist, sodium-glucose cotransporter 1 inhibitor, and loop diuretic), clinical symptoms of heart failure (New York Heart Association class III) were still present. The electrocardiogram (ECG) revealed sinus rhythm with LBBB (QRS interval duration 165 ms), and echocardiography showed severe dysfunction of the left ventricle, with left ventricular ejection fraction (LVEF) 30% and with contraction abnormalities typical for LBBB-related dyssynchrony (apical rocking and septal flash). Global longitudinal strain (GLS) and the performance of the 6-min walk distance (6MWD) were poor (−11.1% and 310 m, respectively). Eventually, the patient qualified for implantation of BiV pacing with defibrillator. The first attempt to implant the device was performed on 31 May 2023, but it was unsuccessful. The retrograde coronary sinus venography revealed its poor bed, and the only available posterolateral branch (Figure 1) had an acute takeoff angle with a double downward angulation in its proximal part. Neither of the two highly experienced operators, despite numbers of attempts and the use of a subselector catheter (Attain Select II, Medtronic, MN, USA), managed to overcome the obstacle and place the lead in the selected location. Finally, a single-chamber implantable cardioverter defibrillator (ICD) for primary prevention of sudden cardiac death, with the defibrillation lead placed in the right ventricular apex, was implanted.

**Figure 2:**
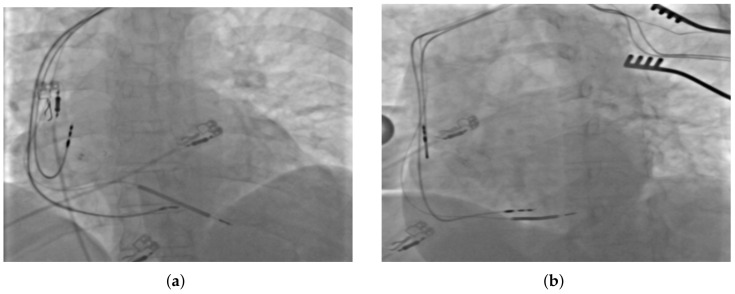
Fluoroscopic image presenting the final placement of all implanted leads in (**a**) antero-posterior, and (**b**) left anterior oblique projections.

However, this approach obviously was not optimal and it did not have a significant impact on the patient’s general condition and her quality of life. Therefore, it was decided to make another attempt to deliver CRT in this patient, this time by CSP. On 3 July 2023, with support of an EP-Tracer recording electrophysiology system, effective implantation of the pacing lead into left bundle branch area was performed, and, thus, narrowing of the QRS (resolution of the LBBB) with effective resynchronization were obtained. During the procedure, a catheter dedicated to placing the lead in the left bundle branch area (Selectra 3D 39-55, Biotronik, Berlin, Germany) and a standard stylet-driven pacing lead (Solia S60, Biotronik) were used. The lead distal tip was introduced into the right ventricle, close to the intraventricular septum area, and short pacing tests monitored with an electrophysiology system enabled the operators to find the most optimal location for its fixation (Figure 2).

**Figure 3:**
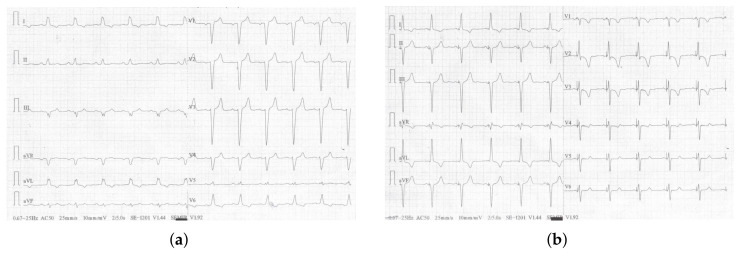
Electrocardiogram showing cardiac electrical activity (**a**) native (without pacing)—QRS duration 160 ms, and (**b**) triggered by left bundle branch area pacing—QRS duration 105 ms.

During prolonged pacing, a lead tip helix was extended deep inside the LBBA—8 turns, which led to immediate resolution of LBBB and significant shortening of the QRS interval (Figure 3). All the leads, the LBBAP lead, the additionally implanted atrial lead (Solia S53, Biotronik), and the high-voltage lead implanted during the previous procedure, were plugged into appropriate ports of a new device (Compia MRI CRT-D, Medtronic). The pacemaker was programmed in the DDD mode (LV only). The patient was discharged from the hospital on the second day after the procedure, in a good general condition, without overt symptoms of heart failure.

**Figure 4:**
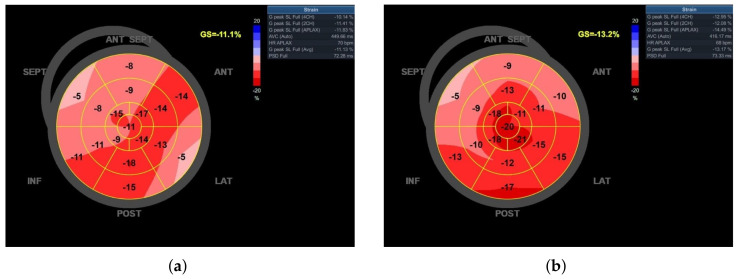
Bull’s eye diagram of left ventricle global longitudinal strain (LV-GLS): (**a**) before, and (**b**) after left bundle branch area pacing (LBBAP).

All final electrophysiological parameters were very good and did not change significantly during the next 6 months of follow-up (Table 1). Twenty-four hours after the procedure, the performance of 6MWD increased by 21%, from 310 to 375 m, and LV-GLS increased by 18.9%, from −11.1 to −13.2% (Figure 4). Left ventricular ejection fraction estimated on the day after the procedure did not change; however, after 6 months of follow-up, it noticeably increased (from 30 to 37%), while the performance of both 6MWD and LV-GLS remained stable. The ventricular pacing percentage at the 6-month follow-up visit was 99.7%.

## 4. Discussion

Cardiac resynchronization electrotherapy has emerged as one of the most spectacular forms of heart failure treatment, a perfect addendum to modern pharmacotherapy. Unfortunately, for various reasons, commonly not sufficiently recognized, more than 30% of eligible patients are not beneficiaries of this treatment method [5]. However, also in this group of patients (non-responders), it is possible to find a solution to overcome the encountered obstacles, to deliver efficient resynchronization, and to reach defined therapeutic goals. This is such a case.

This report demonstrates that if biventricular CRT cannot be achieved due to technical problems in placing the left ventricle pacing lead in the appropriate branch of the coronary sinus, LBBAP may be an effective alternative in patients with HFrEF, even with co-existing LBBB. This form of pacing made it possible to achieve optimal cardiac resynchronization, electrical (resolution of LBBB and QRS interval shortening) and mechanical (LV-GLS and LVEF improvement) improvement, and, most of all, an objective clinical improvement (better performance of 6MWD), maintained after 6 months of follow-up. There are reports presenting that physiological stimulation, especially His bundle pacing, may be an alternative to biventricular pacing [10], but this is not always achievable with co-existing LBBB. Left bundle branch area pacing is a method that can be effectively used, even in distal intraventricular blocks that cannot be corrected with His bundle pacing. This is an obvious advantage of this form of electrotherapy [11].

Before CSP was clinically implemented, patients after a failed attempt of BiV-CRT implantation usually underwent a surgical implantation of epicardial leads. His bundle pacing emerged as a good alternative option, but it has its limitations and is not effective in most patients with heart failure, especially those with co-existing LBBB. Although LBBAP is a quite novel method of cardiac pacing and has been clinically used only for a few years, its popularity is rapidly growing. This is because it offers a significant number of advantages over His bundle pacing, such as better and more stable electrical parameters (including pacing threshold), greater effectiveness in overcoming intraventricular conduction disturbances, and a lower rate of lead-related complications [12]. Moreover, from the technical point of view, it seems to be easier and less time consuming, with a shorter learning curve. One may presume that LBBAP is currently the most friendly and clinically effective cardiac pacing modality. Unfortunately, intuition supported by experience and reason, and even several observational studies, are not enough to consider LBBAP as the optimal cardiac pacing method, and thus randomized trials are necessary.

The up-to-date ESC guidelines on cardiac pacing and cardiac resynchronization therapy emphasize that, in the coming years, the role of CSP will rather increase; however, currently, BiV pacing is still the method of choice in patients eligible for CRT with co-existing LBBB [13]. Right ventricular pacing, which has been the primary cardiac pacing modality for decades, seems to be slowly and gradually losing its importance. While it is true that it is still the primary mode of cardiac pacing all over the world, new modes of cardiac pacing have emerged, and, actually, they may overshadow RVP soon. Data from observational and randomized studies confirm that BiV stimulation is a better pacing modality over RVP, especially in patients with reduced LVEF. The studies show its better impact on LVEF, NYHA class, performance of 6MWT, quality of life, number of hospitalizations, and mortality, when compared with RVP [14,15]. CSP (His bundle or LBBAP) is the next step forward in the development of cardiac electrotherapy. Growing interest is observed in this form of pacing, mostly due to its potential to achieve an optimal hemodynamic response. The implantation technique is more complex, and the support of an electrophysiology recoding system is necessary; however, this pacing modality does not disturb synchronous ventricular contraction in patients with a narrow QRS complex, and, in patients with intraventricular conduction abnormalities, it provides effective resynchronization, and thus practically eliminates the risk of pacemaker-induced cardiomyopathy development [12,16]. Cardiologists proceeding with electrotherapy who commonly use CSP are firmly convinced that this is the best possible form of cardiac pacing, offering a natural advantage over any other pacing modality, including BiV pacing. Moreover, CSP is perceived to be an optimal method of pacing also in patients with conduction disorders, which occur and/or progress after certain structural heart procedures, including transcatheter aortic valve implantation [17].

Nevertheless, there is currently too little hard evidence to support this conviction. A few, rather small randomized trials and only several observational studies presenting that CSP is a better pacing option than BiV pacing are not enough to conclude that this approach is evidence-based [18]. This is why ESC guidelines on cardiac pacing and cardiac resynchronization therapy from 2021 firmly recommend BiV pacing rather than RVP (Class I, level of evidence A) in patients with reduced LVEF (<40%), regardless of QRS complex duration and clinical symptoms of heart failure [13]. Unfortunately, the same ESC guidelines even in symptomatic patients with significantly reduced LVEF do not recommend CSP, either His bundle or LBBAP, as a pacing modality, and this is primarily due to a shortage of hard data confirming the efficiency/advantage of this form of pacing over BiV pacing. His bundle or epicardial pacing are indicated only as bailout options, e.g., in the case of technical problems [13]. However, it is worth emphasizing that the Heart Rhythm Society (HRS) has recently presented guidelines on cardiac physiologic pacing for the avoidance and mitigation of heart failure recommending CSP (both His bundle and LBBAP) in patients with EF 36–50% at the same level of recommendation as BiV pacing (class of recommendation II a), regardless of QRS complex duration [19]. This shows that most of the available research results, even if only observational, with a small number of patients and short duration of follow-up, indicate that, especially in patients with reduced LVEF, CSP may be more optimal than BiV pacing.

There are still too few data to consider LBBAP, even as a good bailout option, especially since there are concerns about the functioning of these leads in the long term, or the feasibility of their extraction. However, there is no doubt that this is a promising method [20]. More and more data prove that CSP is a better form of electrotherapy than RVP [21], and observational studies suggest that, eventually, CSP may also be superior to biventricular CRT [22]. Further research is needed. We need more randomized controlled studies. Some of them are already running, such as the LEFT-BUNDLE-CRT trial or the LeCaRt trial, in which at least 170 patients with an indication for CRT will be randomized to either LBBAP or BiVP. The investigators estimate that the data will be completed at the end of 2024. The results of these studies have a potential to impact future guidelines on cardiac pacing and cardiac resynchronization therapy.

## 5. Conclusions

Our report shows that, in patients with HFrEF and coexisting LBBB, LBBAP may be an effective alternative if technical difficulties are encountered during biventricular CRT implantation. All such cases are helpful to strengthen the belief of cardiac electrotherapy centers that CSP is the proper path of further development and encourage cardiologists to continue research in this area.

## Figures and Tables

**Table 1:** Cardiac electrophysiologic/pacing variables and main clinical characteristics determined before pacemaker implantation (Baseline), the time of implantation (0D), the day after pacemaker implantation (1D), and at the 6 month follow-up visit (6M). Abbreviations: Threshold—pacing capture threshold; R-wave—R-wave sensing; QRSd—QRS complex duration time; V6 RWPT—V6 R-wave peak time; V6-V1 int.p—V6-V1 interpeak interval; 6MWD—6 min walk distance; LV-GLS—left ventricular–global longitudinal strain; LVEF—left ventricular ejection fraction.

	Baseline	0D	1D	6M
Threshold [V/ms]	-	0.5/0.4	0.5/0.4	0.25/0.4
R-wave [mV]	-	14.5	15.6	15.7
QRSd [ms]	165	105	105	105
V6 RWPT [ms]	-	65	-	-
V6-V1 int.p [ms]	-	35	-	-
6MWD [m]	310	-	375	379
LV-GLS [%]	−11.1	-	−13.2	−13.4
LVEF [%]	30	-	30	37

## Data Availability

The data supporting the report are available from Jędrzej Michalik (jedri1616@gmail.com) on reasonable request.
